# Effect of Doping Cement Mortar with Triclosan, Hypochlorous Acid, Silver Nanoparticles and Graphene Oxide on Its Mechanical and Biological Properties

**DOI:** 10.3390/ma17246288

**Published:** 2024-12-23

**Authors:** Mikołaj Paciejewski, Agata Lange, Sławomir Jaworski, Marta Kutwin, Aneta Bombalska, Jarosław Siwiński, Klaudia Olkowicz, Jadwiga Mierczyk, Kamila Narojczyk, Zdzisław Bogdanowicz, Barbara Nasiłowska

**Affiliations:** 1 Faculty of Mechanical Engineering, Military University of Technology, gen. S. Kaliskiego 2, 00-908 Warsaw, Poland; 2 Department of Nanobiotechnology, Institute of Biology, Warsaw University of Life Sciences, Ciszewskiego 8, 02-786 Warsaw, Poland; 3 Institute of Optoelectronics, Military University of Technology, gen. S. Kaliskiego 2, 00-908 Warsaw, Poland; 4 Faculty of Civil Engineering and Geodesy, Military University of Technology, gen. S. Kaliskiego 2, 00-908 Warsaw, Poland; 5 Aircraft Airworthiness Division, Air Force Institute of Technology, 01-494 Warsaw, Poland

**Keywords:** graphene oxide, cement mortar, silver nanoparticles, hypochlorous acid, triclosan

## Abstract

In order to improve the performance of cement mortar (Portland cement), it was enriched with triclosan, hypochlorous acid, silver nanoparticles and graphene oxide. Cement mortar is used, among other things, to fill the gaps between the tiles of building porcelain stoneware. A number of structural, mechanical and biological tests were carried out. The structural tests included microscopic analysis and contact angle, reflectance and IR spectra, while the mechanical tests involved static bending and compression testing. These tests showed that the additions of graphene oxide and hypochlorous acid were most beneficial. These additions, although not detected by spectral methods, resulted in a significant increase in contact angle and mechanical properties. Studies of the viability of the bacteria Pseudomonas aeruginosa and Staphylococcus aureus showed that all the additives used resulted in a decrease in viability compared to the undoped cement mortar. There was also a beneficial decrease in the viability of fungi of the genus Fusarium on cement mortar mainly doped with silver nanoparticles.

## 1. Introduction

When renovating long-standing buildings as well as flooded buildings, the walls in rooms that are exposed to harmful agents such as bacteria or fungi after plaster removal should be treated with a coating that neutralizes the presence of harmful pathogens living in cracks and pores. This is particularly important not only in domestic premises, but also in public areas such as shelters and hospitals.

Although bacterial biofilms mainly occur on medical devices and are associated with the presence of moisture [1,2], biofilms on dry surfaces have recently been discovered [3,4]. These dry biofilms may contain viable multiresistant organisms, occurring despite the cleaning of clinical surfaces in intensive care units [3,4].

The cementitious mortar that fills the spaces between the tiles of building porcelain stoneware is also exposed to a number of adverse factors that promote its degradation. The main one, however, is moisture, which can not only promote the growth of microorganisms, including bacteria and fungi, but can also lead to a decrease in functional properties, including mechanical properties.

The introduction of the addition of graphene or its derivatives to composites has been the result of many research works [5,6,7,8]. The results of fatigue life studies presented in articles by Demir [6], Rafiee [7] and Li [8], among others, have shown that a small addition of graphene or its derivatives throughout the composite results in improved mechanical properties.

Li et al. [8] observed that the addition of wt. 0.075% multilayer graphene to concrete fills voids in the pore structure, leading to an increase in fatigue life of up to 49.3%. In contrast, the content of ~0.2% graphene in a composite consisting of epoxy fibres increases flexural fatigue life by up to 1200 times [7]. Similarly, Yavari and his team [9] showed that the addition of graphene to a composite resulted in an ∼3–5-fold increase in fatigue life.

In a publication by Liu et al. [10], the mechanic properties of graphene oxide, including Young’s modulus, were determined using calculations. Analysis of structural models of graphene oxide showed that the ordered structure has a higher strength and Young’s modulus (E = 380 ÷ 470 GPa) than the amorphous structure (E = 290 ÷ 430 GPa). This is mainly due to defects in the sp2 carbon network of graphene oxides.

The analysis of the influence of graphene nanostructures in building composites on performance properties such as degradation process (ageing) or static bending, compression and flexural strengths was the subject of research in [11,12,13].

Zeng et al. [11] presented the degradation mechanism of GO-modified mortars under real field conditions. They analyzed the long-term degradation (over 24 months) of GO-modified mortars exposed to sulphate solutions at different concentrations: 0% (reference), 2.1% (field conditions), 5% (laboratory conditions) and 15% (high concentration conditions). Under real-world conditions, the degradation of cement mortar mainly involves the precipitation of gypsum. They showed that well-dispersed GO nanosheets can significantly increase the durability of cementitious materials against sulphate exposure.

Singh and his team [12] revealed that even a small addition of 0.1% graphene to concrete and cement mortar leads to a noticeable significant increase in compressive strength (CS), flexural strength (TS) and flexural strength (FS), up to 10–15%, 20–25% and 20–30%, respectively. The review also highlighted technological challenges such as scalability, economic viability and regulatory compliance for the introduction of graphene nanostructures.

In contrast, Compton et al. in a publication [14] reported on the mechanic properties of graphene paper made from graphene oxide, which had a Young’s modulus of E = 25.6÷32 GPa and a flexural strength limit of Rm = 81.9 ÷ 130 MPa [15,16]. It is interesting to note that non-functionalized graphene oxide typically exhibits bacteriostatic properties [17], but can provide a platform for the gradual release of other substances e.g., bactericidal. A prolonged release of substances with dedicated properties is often desirable, especially if the product is to act in an appropriate long-term manner.

The use of not only graphene, but also other nanocomposites, e.g., silver, in concrete structures or cement mortar can lead to the filling of voids and thus to an increase in static strength. In addition, silver nanoparticles are bactericidal [18,19] and are therefore widely used as a disinfectant in the production of dressing materials, such as bandages, dressings and surgical masks.

There are many literature reports on the bacteriocidal effects of silver nanoparticles [18,19], hypochlorous acid [20] and triclosan [21,22,23]. The other point of view is to investigate the mechanical and structural properties of modified cement mortar with above mentioned specimens together with graphene oxide.

The aim of this publication is to present the results of tests on cement mortar with triclosan, hypochlorous acid, silver nanoparticles and graphene oxide, which, in addition to covering entire surfaces, can also act as a cement mortar for filling gaps between ceramic tiles that is resistant to bacteria and fungi and has enhanced strength. Improving the performance of cement mortar that is particularly exposed to harmful pathogens is important in public areas.

## 2. Materials and Methods

### 2.1. Method of Making Cement Mortar

The subject of the research presented in this publication is cement mortar, the precursor of which was Portland cement, manufactured by Henkel (Düsseldorf, Germany) (CERESIT1355568).

Triclosan (72779-25G-F) from Merck KGaA, Darmstadt, Germany, due to its dry form (powder), was mixed in ultrapure water (concentration 0.0108 g/L) using a Vortex stirrer (10,000 rpm, temp. 22 °C, BioSan, Riga, Latvia). In contrast, admixtures in the form of suspensions were implemented into the cement mortar at the concentrations specified by the manufacturers. A dispersed graphene oxide suspension with a concentration of 4.5 g/L was produced and purchased from the Lukasiewicz Research Network—Institute of Electronic Materials Technology. Silver nanoparticles were from NANO-TECH Poland, Warsaw, Poland, and hypochlorous acid at a concentration of 2000 ppm was supplied by BioMedAqua Sp. z o.o., Dębica, Poland.

To make the cement mortar samples, 20 kg of Portland cement was mixed with 5 L of ultrapure water using a Yato YG 03029 automatic mixer (TOYA S.A. Poland, Wrocław, Poland), mode 2 (155 rpm). The mixing time of the mixture was 5 min. Subsequently, 1 kg of ready-mixed cement mortar was measured, to which the admixture was added at the appropriate concentration (Table 1).

The cement mortar was admixed with triclosan, hypochlorous acid, silver particles and graphene oxide according to the proportions shown in Table 1.

In addition, a sample of C + HClO* was produced by mixing 1 kg of Portland cement, using no water but 0.25 L of 2000 ppm hypochlorous acid.

**Figure 1:**
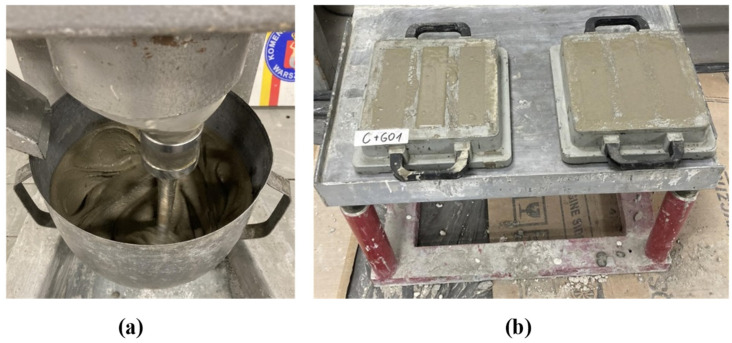
Sample preparation process (**a**) combining ingredients in an ML-LA type laboratory mixer, (**b**) WAM Group OLI Modolla vibrating platform.

After the admixtures were introduced, the ingredients were combined in an ML-LA type laboratory stirrer, mode 1 (140 rpm) (Figure 1a). The mixing time was also 5 min. The resulting cement mortar was placed in 40 × 40 × 160 mm molds. In the next step, these molds were vibrated using a WAM Group OLI Modolla platform (WAMGROUP SPA, Villa Motta, Italy), with a rotational power of 3440 RPM (Figure 1b).

The samples were prepared in 40 × 40 × 160 mm. After 24 h, samples were disassembled and cured in water at 20 °C in accordance with [24]. The mixing was executed under laboratory conditions with dried aggregates and powder materials. The room temperature was maintained at around 20 °C during mixing and testing. After curing for 28 days, the samples were tested for compressive strength, in accordance with the standard [25], and flexural strength, accordance with the standard [26], using a MEGA 6-3000-150 (Form+Test, Riedlingen, Germany) hydraulic press.

In addition, flat specimens, measuring approximately 50 × 50 × 5 mm and approximately 10 × 10 × 5 mm, respectively, were prepared for further structural and biological tests.

### 2.2. Research Methods

#### 2.2.1. Structural Testing of Surfaces

- Stereoscopic microscope:
  Structure imaging of cement mortar, doped with triclosan (C + Tr, C + GO +Tr, C + HClO + Tr, C + Tr + Ag and C + GO + HClO+ Tr + Ag), hypochlorous acid (C + HClO, C + HClO*, C + GO + HClO, C + HClO + Tr, C + HClO + Ag, C + Tr + Ag and C + GO + HClO+ Tr + Ag), silver nanoparticles (C + Ag, C + GO + HClO, C + GO + Ag, C + HClO + Ag, C + Tr + Ag and C + GO + HClO+ Tr + Ag) and graphene oxide (C + GO, C + GO + HClO, C + GO +Tr, C + GO + Ag and C + GO + HClO+ Tr + Ag) was performed using a Zeiss SmartZoom 5 steerable microscope (Carl Zeiss Meditec AG (Headquarter) Göschwitzer, Jena, Germany). A PlanApo D 1.6/0.1 FWD 36 mm objective was used.
- Contact angle:
  The contact angle was measured using an optical microscope (6000 VHX, Keyence Corporation, Osaka, Japan). Droplets of ultrapure water with a volume of about 3 µL from a constant height of 5 mm were dropped onto the surface of a laboratory slide with C, C + GO, C + HClO, C + HClO*, C + Tr, C + Ag, C + GO + HClO, C + GO +Tr, C + GO + Ag, C + HClO + Tr, C + HClO + Ag, C + Tr + Ag and C + GO + HClO+ Tr + Ag.
- Fourier-transform Infrared Spectroscopy (FTIR) study of the chemical surface composition:
  Samples listed in Table 1 were analyzed by FTIR (Nicolet IS50, FTIR, ThermoFisher SCIENTIFIC, Waltham, MA, USA) using ATR mode in a range of 400–4000 cm^−1^ with a resolution of 4 cm^−1^ and 64 scans.
- Reflectance:
  Reflectance measurements were carried out on a Lambda 900 spectrometer from Perkin Elmer (Waltham, MA, USA), equipped with a PELA 1001 Downward Viewing integration sphere from Labsphere (North Sutton, NH, USA), which allows measurement of total reflectance, diffuse reflectance and determination of specular reflectance. Total reflectance values were determined in the spectral range 250–2500 nm with a measurement step of 1 nm. The measurement results are the dependence of the total reflectance R [%] on the wavelength λ [nm].

#### 2.2.2. Tests of Mechanical Properties

**Figure 2:**
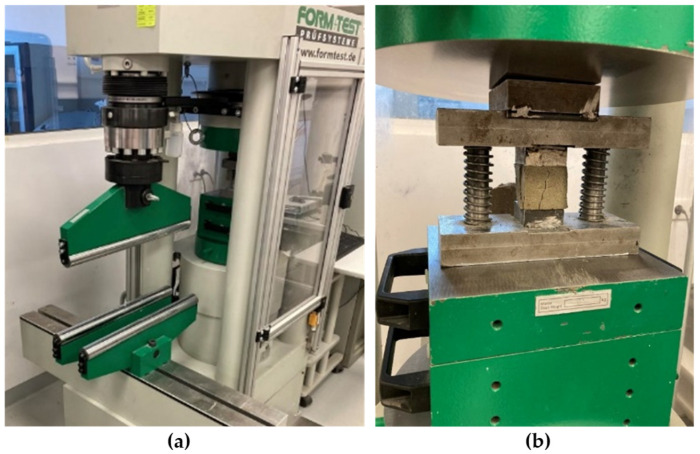
Form Test Mega 6 machine with single-point load beam flexural strength test attachment (**a**) and compression test attachment (**b**).

After 28 days of maturation of the beams in tap water (Section 2.1) according to standard [24,25,26], tests were carried out on the mechanical properties for bending with single-point loading of the cement mortar beams (Figure 2a) and for compression (Figure 2b). These tests were carried out using a Form Test Mega 6 hydraulic machine.

#### 2.2.3. Biological Research

- Analysis of bacterial inhibition zones:
  Analysis of bacterial growth and its inhibition after exposure to cement mortar was prepared with two bacterial strains: *Staphylococcus aureus* (ATCC 25923) and *Pseudomonas aeruginosa* (ATCC 27853), which were obtained from the American Type Culture Collection (ATCC) and they were maintained in 20% (*v*/*v*) glycerol at −20 °C. On the surface of solidified Mueller–Hinton agar prepared in Petri dishes (Ø90 mm), the spread plate method of bacterial suspension (0.5 on the McFarland scale) was performed, and then the samples of cement mortar (10 × 10 mm) were placed on the surface. The plates were incubated in standard conditions (24 h, 37 °C). After incubation, the inhibition zones were measured and captured with an Azure C400 (Azure Biosystem, Dublin, CA, USA). The procedure of preparing samples to this analysis was described in [14].
- Bacterial viability analysis:
  Bacterial viability of two tested bacteria species (*S. aureus* and *P. aeruginosa*) exposed to cement mortar was analyzed using an XTT Cell Proliferation Kit II (cat no. 11465015001, Merck, Darmstadt, Germany). Samples were placed in the wells of a 24-well plate, in which 1 mL of Mueller–Hinton broth (Biomaxima, Lublin, Poland) was added and each well was inoculated with 10 µL bacteria suspensions (1.5 × 10^8^ cells/mL). After 24 h of incubation (37 °C), XTT reagent was added and after 30 min, absorbance with an excitation wavelength of 450 nm and emission of 690 nm was measured. The results are presented as a viability % relative to the control. The procedure of preparing samples to this analysis was described in [14].
- Fungi growth analysis:
  The analysis of samples listed in Table 1 on the growth of fungi from the genus Fusarium was performed. For this purpose, cement mortar samples were soaked with fungi spores suspension (10^6^ cells/mL) and prepared samples were placed onto Sabouard agar (Biomaxima, Lublin, Poland). Plates were incubated (25 °C) for 7 and 14 days. Fungi growth was measured and captured with an Azure C400 camera (Azure Biosystem, Dublin, CA, USA).

## 3. Results

### 3.1. Structural Research

#### 3.1.1. Stereoscopic Microscope

**Figure 3:**
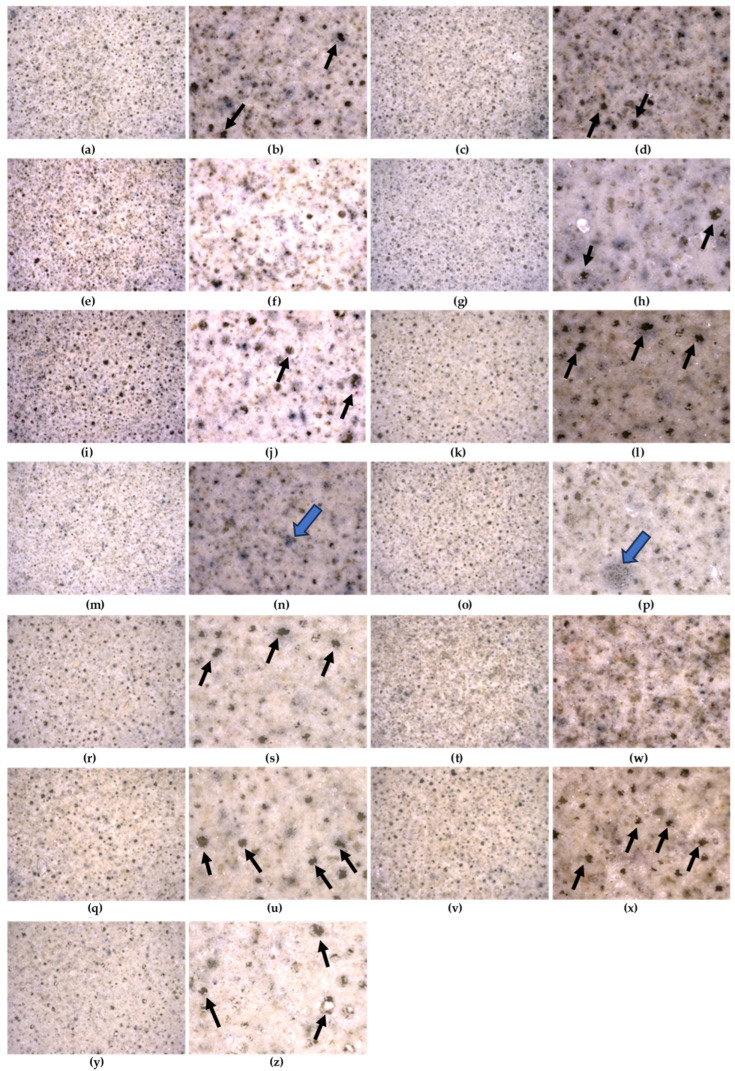
Surface area of samples C (**a**,**b**), C + GO (**c**,**d**), C + HClO (**e**,**f**), C + HClO* (**g**,**h**), C + Tr (**i**,**j**), C + Ag (**k**,**l**), C + GO + HClO (**m**,**n**), C + GO +Tr (**o**,**p**), C + GO + Ag (**r**,**s**), C + HClO + Tr (**t**,**w**), C + HClO + Ag (**q**,**u**), C + Tr + Ag (**v**,**x**) and C + GO + HClO+ Tr + Ag (**y**,**z**), taken with a stereoscopic microscope at pow. 35× (**a**,**c**,**e**,**g**,**i**,**k**,**m**,**o**,**r**,**t**,**q**,**v**,**y**) and 100× (**b**,**d**,**f**,**h**,**j**,**l**,**n**,**p**,**s**,**w**,**u**,**x**,**z**).

In Figure 3, images of cement mortar C (Figure 3a,b) with the respective additives, i.e., triclosan (Tr), hypochlorous acid (HClO), silver particles (Ag) and graphene oxide (GO), are shown; C + GO (Figure 3c,d), C + HClO (Figure 3e,f), C + HClO* (Figure 3g,h), C + Tr (Figure 3i,j), C + Ag (Figure 3k,l), C + GO + HClO (Figure 3m,n), C + GO +Tr (Figure 3o,p), C + GO + Ag (Figure 3r,s), C + HClO + Tr (Figure 3t,w), C + HClO + Ag (Figure 3q,u), C + Tr + Ag (Figure 3v,x) and C + GO + HClO+ Tr + Ag (Figure 3y,z). Images were taken using a digital stereo microscope at 35× (Figure 3a,c,e,g,i,k,m,o,q,r,t,v,y) and 100× (Figure 3b,d,f,h,j,l,n,p,s,w,u,x,z). All samples had a granular structure, typical of samples containing cement. This structure is evident for the analyzed magnifications of 35 and 100×.

It should be noted, in samples C (Figure 3b), C + GO (Figure 3d), C + HClO* (Figure 3h), C + Tr (Figure 3j), C + Ag (Figure 3l), C + GO + Ag (Figure 3s), C + HClO + Ag (Figure 3u), C + Tr + Ag (Figure 3x) and C + GO + HClO+ Tr + Ag (Figure 3z), micropores, indicated by black arrows, are visible. Their occurrence may be due to insufficiently long exposure times to vibration or the amount of liquid admixtures that influenced the density of the mixture.

In contrast to the C + GO sample (Figure 3d), analysis of the surface structure of the cement mortar C + GO + HClO (Figure 3n), C + GO +Tr (Figure 3p) revealed visible agglomerations of graphene oxide nanoparticles (marked with a blue arrow).

#### 3.1.2. Contact Angle

**Figure 4:**
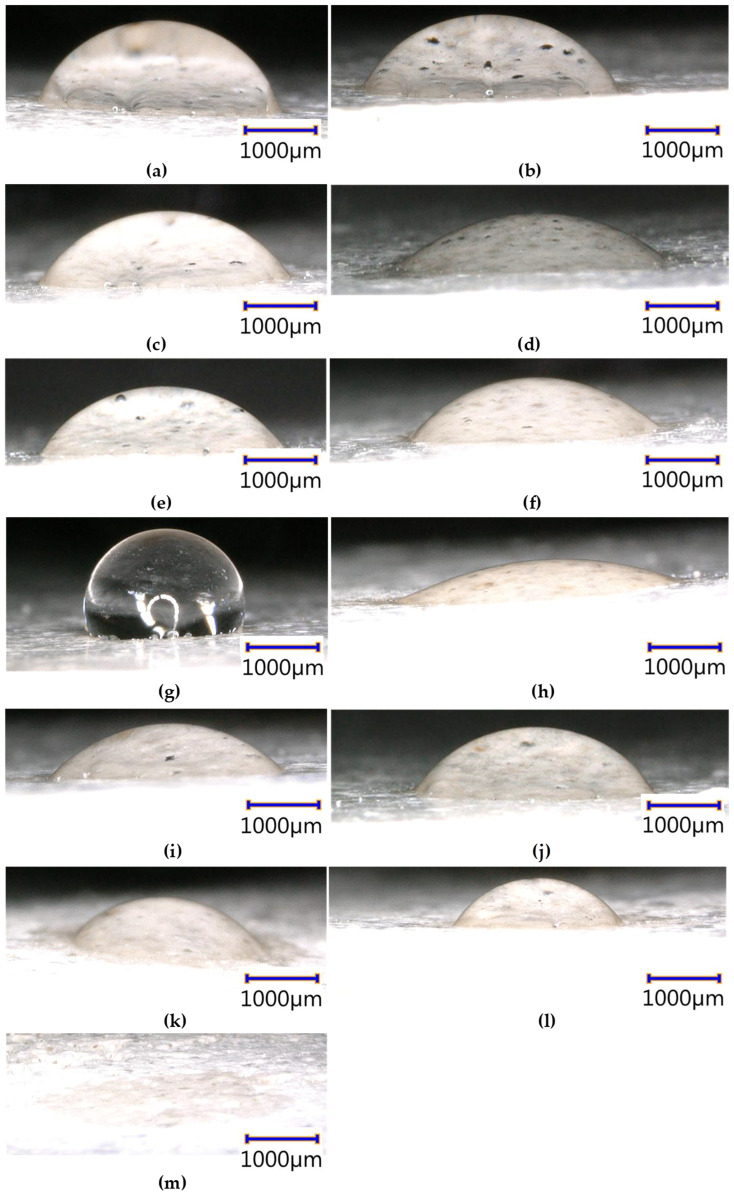
The water droplet applied to the surface of C (**a**), C + GO (**b**), C + HClO (**c**), C + HClO* (**d**), C + Tr (**e**), C + Ag (**f**) samples, C + GO + HClO (**g**), C + GO +Tr (**h**), C + GO + Ag (**i**), C + HClO + Tr (**j**), C + HClO + Ag (**k**), C + Tr + Ag (**l**) and C + GO + HClO+ Tr + Ag (**m**).

The results of the contact angle tests are presented in Figure 4a–m and Table 2. It is interesting to note that doping the cement mortar with triclosan, hypochlorous acid, silver nanoparticles and graphene oxide significantly influenced the surface contact angle. The results of the tests for the subsequent test groups (even though they were repeated 5× for each treatment) were significantly different. In the case of the C + GO + HClO+ Tr + Ag (Figure 4m) sample, the droplet was gradually absorbed after application and after approximately 1–2 s, the contact angle was 0 deg. The surface of these sample was superhydrophilic. A similar situation occurred for the samples C + GO + Tr (Figure 4h), C + GO + Ag (Figure 4i), C + Tr + Ag (Figure 4l), C + HClO + Ag (Figure 4k) and C + HClO* (Figure 4d) with the difference that the absorption of the droplet occurred after 10 s, 15–20 s, 20 s, 40 s and 60 s respectively. After this time, the contact angle was 0°. The highest contact angle was observed for the C + GO + HClO samples of 82° ± 20°.

**Figure 5:**
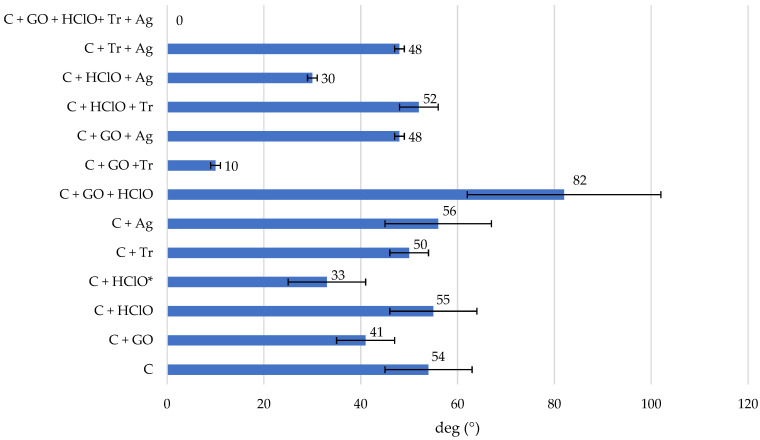
Column diagram of the contact angle of ultrapure water droplets applied to the surface of C, C + GO, C + HClO, C + HClO*, C + Tr, C + Ag, C + GO + HClO, C + GO +Tr, C + GO + Ag, C + HClO + Tr, C + HClO + Ag, C + Tr + Ag and C + GO + HClO+ Tr + Ag samples.

In order to illustrate the differences between the contact angle of the tested cement mortar surfaces after doping with triclosan, hypochlorous acid, silver nanoparticles and graphene oxide, a column graph is shown in Figure 5. A clear increase in the contact angle of the surfaces of the C + GO + HClO samples was observed relative to the other C, C + GO, C + HClO, C + HClO*, C + Tr, C + Ag, C + GO +Tr, C + GO + Ag, C + HClO + Tr, C + HClO + Ag, C + Tr + Ag and C + GO + HClO+ Tr + Ag samples tested.

#### 3.1.3. FTIR

**Figure 6:**
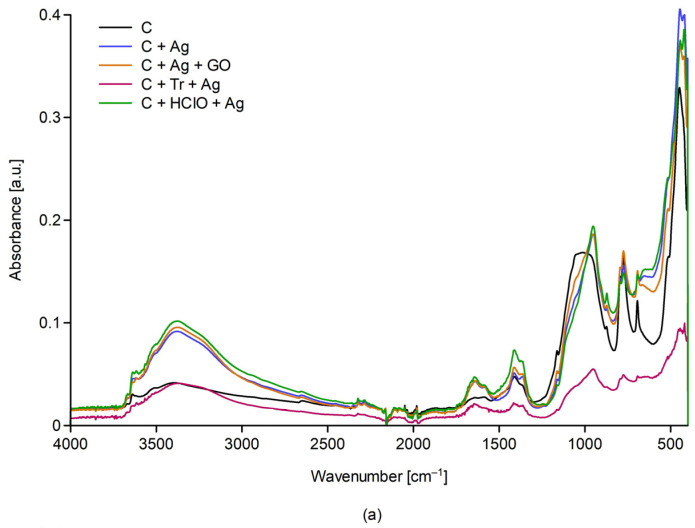

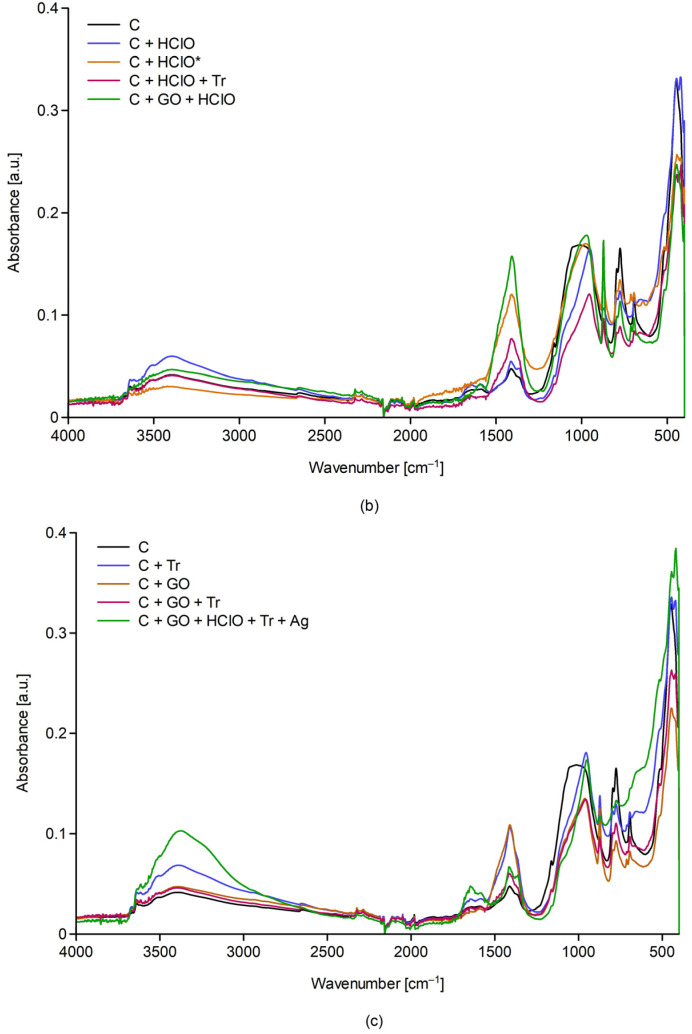
The figure shows FTIR spectra for; (**a**) samples C, C + Ag, C + Ag + GO, C+ Tr + Ag, C+ HClO + Ag; (**b**) samples C, C + HClO, C + HClO*, C + HClO + Tr, C + HClO + GO; (**c**) samples C, C +Tr, C + GO, C + GO + Tr, C + HClO + Tr + Ag + GO (c).

The figure shows the FTIR spectra for samples C, C + Ag, C + Ag + GO, C+ Tr + Ag, C+ HClO + Ag (Figure 6a), C, C + HClO, C + HClO*, C + HClO + Tr, C + HClO + GO (Figure 6b), C, C +Tr, C + GO, C + GO + Tr, C + HClO + Tr + Ag + GO (Figure 6c).

The FTIR analysis was designed to attempt to detect admixtures added to the cement mortar. However, the low concentrations of additives and the inorganic matrix do not allow an accurate chemical analysis of the composites obtained, nor of their possible influence on the formation of the C-S-H phase (C-CaO, S-SiO_2_, H-H_2_O). OH stretching vibrations with SiOH give bands in the same range as in alcohols, and Si-O stretching vibrations extending in the range of 830–1110 cm^−1^ have absorbance values at a similar value for all samples tested. A noticeably variable area is the range 1250–1550 cm^−1^. The absorbance is the lowest for the group of compounds shown in Figure 6a, with the highest absorbance recorded for the composites in the group (Figure 6b) and for the C + GO and C + Tr samples in the group (Figure 6c). Given the chemical nature of the dopants, it can be surmised that this is an effect of the oxidants (e.g., HClO) and oxygen group donors (GO) that they have on -OH bond formation. However, the valence vibration region of the OH groups is virtually invariant for all composites.

#### 3.1.4. Total Reflection—Reflectance

**Figure 7:**
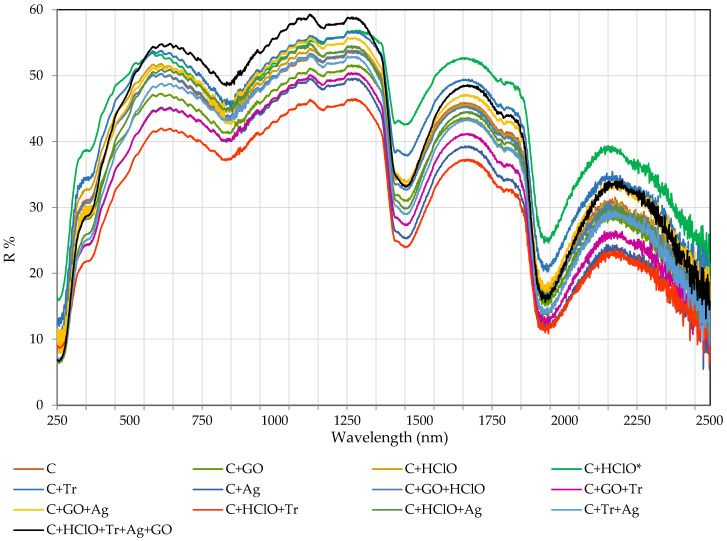
Graphs showing the dependence of reflectance R [%] as a function of wavelength [nm] for samples C, C + GO, C + HClO, C + HClO*, C + Tr, C + Ag, C + GO + HClO, C + GO + Tr, C + GO + Ag, C + HClO + Tr, C + HClO + Ag, C + Tr + Ag, and C + GO + HClO + Tr + Ag.

Figure 7 shows the spectra of total reflectance R (reflectance) for samples C, C + GO, C + HClO, C + HClO*, C + Tr, C + Ag, C + GO + HClO, C + GO +Tr, C + GO + Ag, C + HClO + Tr, C + HClO + Ag, C + Tr + Ag and C + GO + HClO+ Tr + Ag.

The decisive influence on the value of the measured total reflectance R (reflectance) is the type of surface of the test sample. A rough surface will show a higher reflectance value. The shape of the reflectance characteristics of the samples is very similar, differing only in the magnitude of the reflectance value (R%). The very similar shape of the characteristics in Figure 7 may be influenced by the ‘base’ of the samples. All samples consisted of cement mortar. The samples differed in the admixtures (triclosan, hypochlorous acid, silver nanoparticles and graphene oxide), which caused a slight change in the colour of the sample (Figure 3) and a change in the type of sample surface (micro pores appearing). The C + HClO + Tr + Ag + GO sample shows the highest reflectance value (approx. 60%) in the 550–650 nm (approx. 54%) and 600–1250 nm ranges, and the C + HClO* sample in the 1625–1750 nm range (approx. 52%). The other samples have the same shape but show lower reflectance values.

### 3.2. Testing the Flexural and Compressive Strength of Cement Mortar Beams

**Figure 8:**
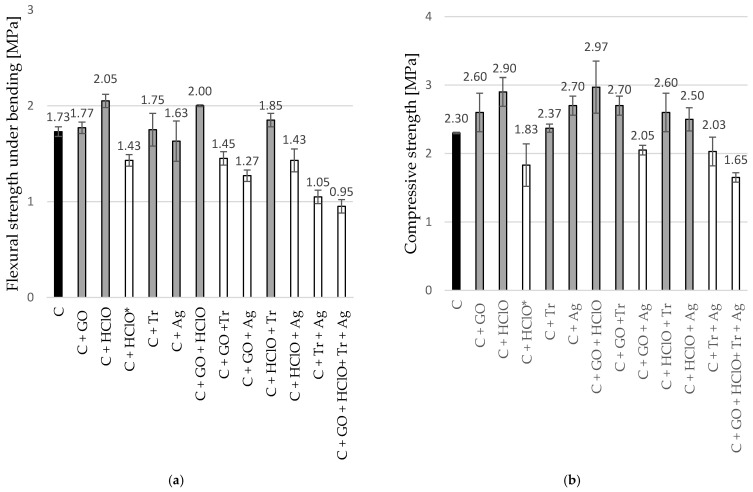
Bar charts of single-point bending (fracture) (**a**) and compression (**b**) tests for samples C, C + GO, C + HClO, C + HClO*, C + Tr, C + Ag, C + GO + HClO, C + GO + Tr, C + GO + Ag, C + HClO + Tr, C + HClO + Ag, C + Tr + Ag, and C + GO + HClO + Tr + Ag.

The results of the single-point loading flexural strength test for cement mortar beams and the compressive strength tests are presented in Table 3 and Figure 8. These tests covered samples C, C + GO, C + HClO, C + HClO*, C + Tr, C + Ag, C + GO + HClO, C + GO + Tr, C + GO + Ag, C + HClO + Tr, C + HClO + Ag, C + Tr + Ag, and C + GO + HClO + Tr + Ag.

An increase in the amount of liquid admixtures, as expected, led to a deterioration of interstructural bonds and mechanical strength parameters. An exception was the addition of hypochlorous acid in amounts of 5.1–10.2% of the added water, which resulted in an increase in the flexural strength of the cement mortar by 21.2–18.2% (C + HClO) and an increase in compressive strength by 26.0 and 29% (C + GO + HClO), respectively. Smaller strength improvements were observed in other series with the addition of hypochlorous acid to the water. This is related to the formation of micropores in the granular structure of the sample, which, by creating microvoids, globally weaken the entire specimen.

This study also demonstrated that the use of graphene oxide enhanced the strength properties of the cement mortar. During compression tests, conclusions analogous to those from the bending tests were observed. A thinner mixture containing more additives, during the binding and drying process, led to the formation of more micropores and voids, which weakened the tested material. Of note is the structural reinforcement achieved by adding graphene oxide to the mixture. The test results showed improved mechanical properties for the samples C + GO, C + HClO, C + GO + HClO, and C + HClO + Tr compared to the control sample C.

### 3.3. Badania Biologiczne Biological Studies

#### 3.3.1. Results of Bacterial Growth Inhibition Tests

**Figure 9:**
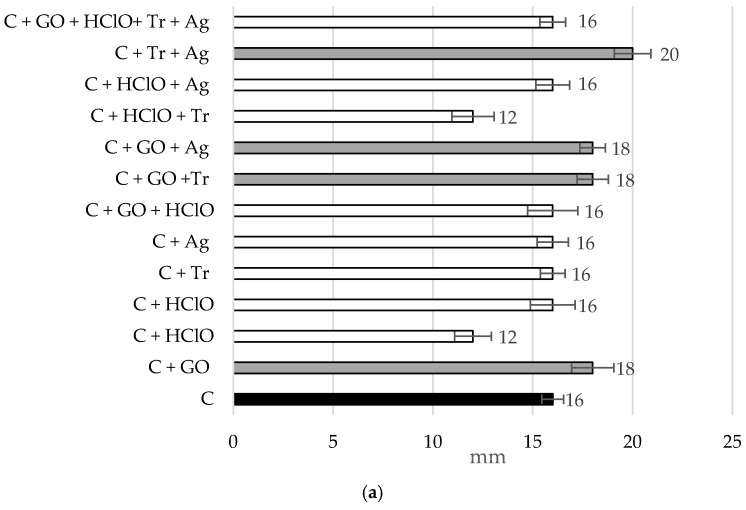

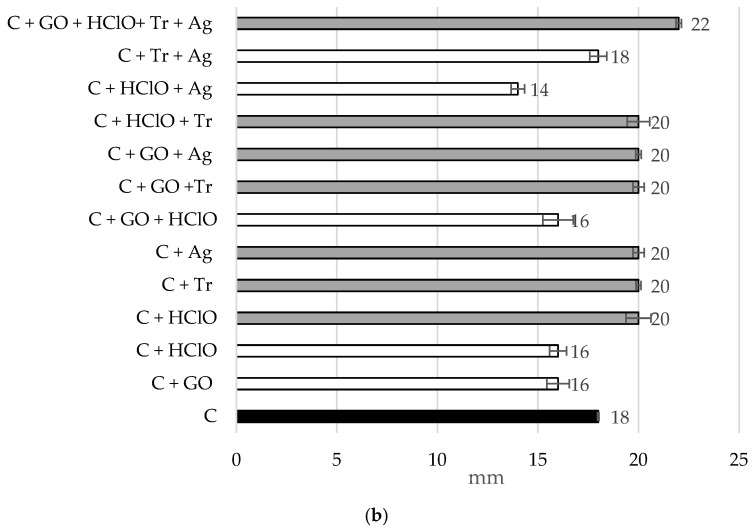
Bar charts showing bacterial growth inhibition [mm] for *Pseudomonas aeruginosa* (**a**) and *Staphylococcus aureus* (**b**) for samples C, C + GO, C + HClO, C + HClO*, C + Tr, C + Ag, C + GO + HClO, C + GO + Tr, C + GO + Ag, C + HClO + Tr, C + HClO + Ag, C + Tr + Ag, and C + GO + HClO + Tr + Ag.

Figure 9 presents the results of bacterial growth inhibition tests for Pseudomonas aeruginosa and Staphylococcus aureus for samples C, C + GO, C + HClO, C + HClO*, C + Tr, C + Ag, C + GO + HClO, C + GO + Tr, C + GO + Ag, C + HClO + Tr, C + HClO + Ag, C + Tr + Ag, and C + GO + HClO + Tr + Ag.

It was observed that the sample C + HClO + Ag exhibited the highest resistance to Staphylococcus aureus, while samples C + HClO and C + HClO + Tr were most effective against Pseudomonas aeruginosa.

Interestingly, the cement mortar itself demonstrated a degree of bacterial growth inhibition for both *Pseudomonas aeruginosa* and *Staphylococcus aureus*.

To illustrate the improvement in bactericidal properties: black bars represent the cement mortar alone (C). White bars indicate the inhibition zones where the application of specific additives enhanced the beneficial effect. Gray bars represent results without a significant improvement.

#### 3.3.2. Results of Bacterial Viability Tests

**Figure 10:**
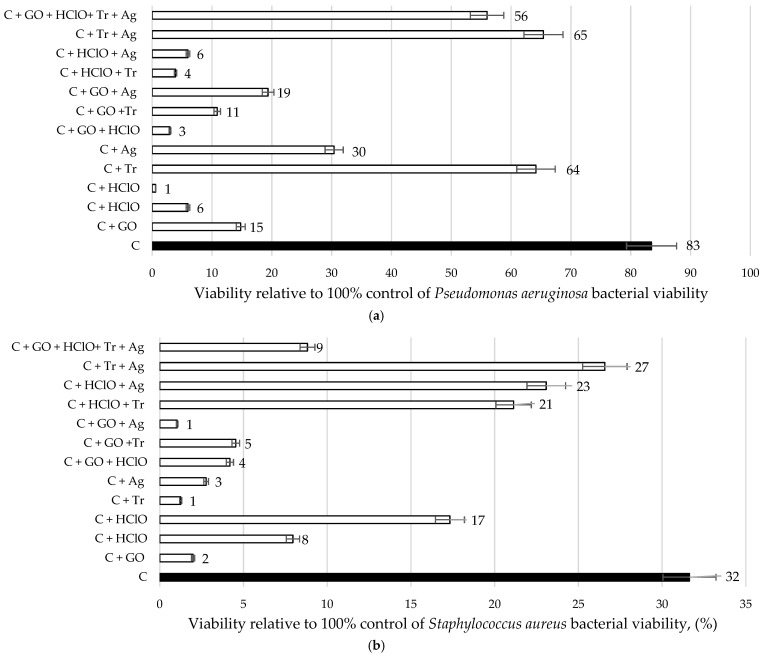
Bar charts of bacterial viability for *Pseudomonas aeruginosa* (**a**) and *Staphylococcus aureus* (**b**) for samples C, C + GO, C + HClO, C + HClO*, C + Tr, C + Ag, C + GO + HClO, C + GO + Tr, C + GO + Ag, C + HClO + Tr, C + HClO + Ag, C + Tr + Ag, and C + GO + HClO + Tr + Ag, expressed relative to 100% control.

Figure 10 presents the results of bacterial viability tests for *Pseudomonas aeruginosa* and *Staphylococcus aureus* for sample C and samples C + GO, C + HClO, C + HClO*, C + Tr, C + Ag, C + GO + HClO, C + GO + Tr, C + GO + Ag, C + HClO + Tr, C + HClO + Ag, C + Tr + Ag, and C + GO + HClO + Tr + Ag. The bacterial viability was compared to parallel cultures of *Pseudomonas aeruginosa* and *Staphylococcus aureus* grown without the addition of cement mortar.

It was observed that cement mortar alone exhibits bactericidal properties. However, this effect was enhanced with the use of specific additives.

For *Staphylococcus aureus*, a significant reduction in viability was noted, particularly in samples containing triclosan and hypochlorous acid. *Pseudomonas aeruginosa* also showed susceptibility to the hypochlorous acid, triclosan and silver nanoparticles used in the samples.

Similar to Section 3.3.1, black bars represent the cement mortar alone (C), while white bars indicate the viability of the tested bacteria. It was observed that the application of the studied additives strengthened the bactericidal effect.

#### 3.3.3. Results of Fungal Growth Inhibition Tests

**Figure 11:**
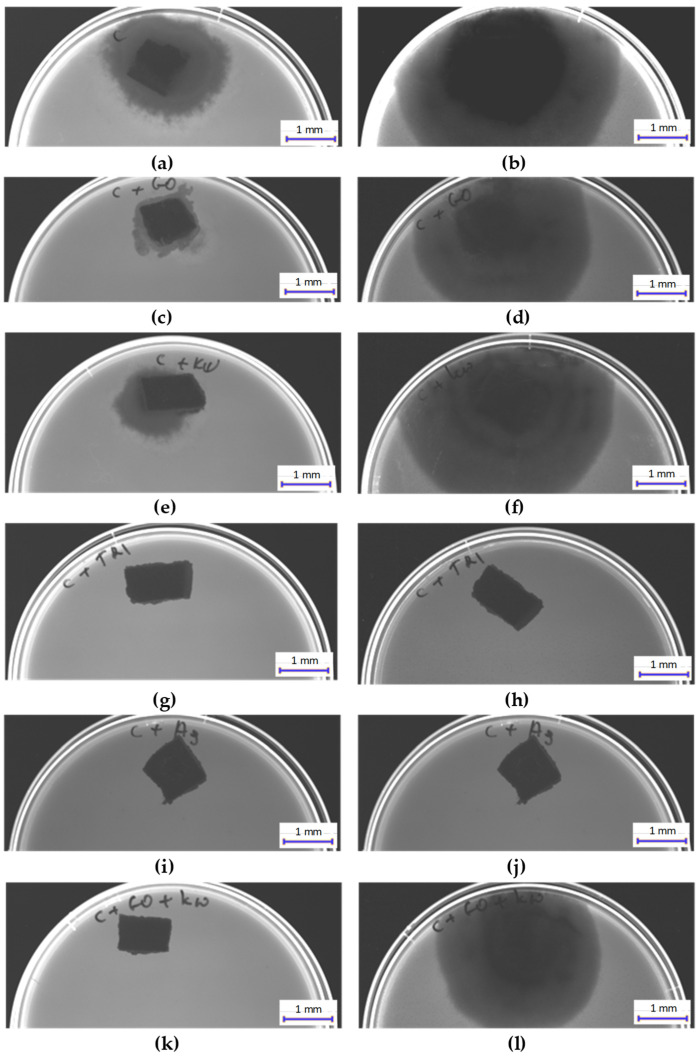
Images showing the degree of *Fusarium* growth inhibition after 7 (**a**,**c**,**e**,**g**,**i**,**k**) and 14 (**b**,**d**,**h**,**j**,**l**) days on the surface of samples C (**a**,**b**), C + GO (**c**,**d**), C + HClO (**e**,**f**), C + Tr (**g**,**h**), C + Ag (**i**,**j**), C + GO + HClO (**k**,**l**).

**Figure 12:**
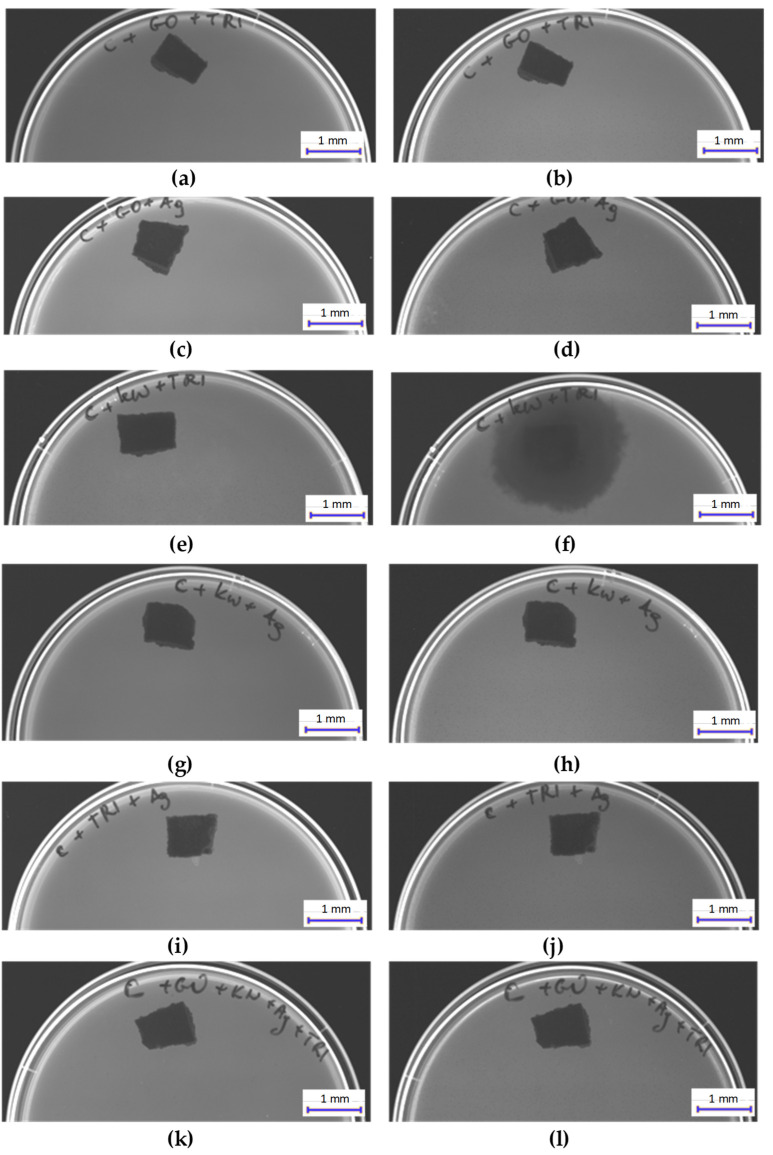
Images showing the degree of *Fusarium* growth inhibition after 7 (**a**,**c**,**e**,**g**,**i**,**k**) and 14 (**b**,**d**,**h**,**j**,**l**) days on the surface of samples C + GO + Tr (**a**,**b**), C + GO + Ag (**c**,**d**), C + HClO + Tr (**e**,**f**), C + HClO + Ag (**g**,**h**), C + Tr + Ag (**i**,**j**), and C + GO + HClO + Tr + Ag (**k**,**l**).

Figure 11 and Figure 12 present the results of fungal growth inhibition tests for *Fusarium* after 7 days (Figure 11) and 14 days (Figure 12), cultured on samples C, C + GO, C + HClO, C + HClO*, C + Tr, C + Ag, C + GO + HClO, C + GO + Tr, C + GO + Ag, C + HClO + Tr, C + HClO + Ag, C + Tr + Ag, and C + GO + HClO + Tr + Ag.

The surfaces most resistant to fungal activity were observed in samples C + Ag, C + Tr, C + GO + HClO, C + GO + Tr, C + GO + Ag, C + HClO + Ag, C + Tr + Ag, and C + GO + HClO + Tr + Ag.

## 4. Discussion

Currently, the most commonly used additives in cement mortar are mainly microsilicas and plasticizers, which aim to reduce water absorption. Such additives aim to improve the functional properties, particularly mechanical strength. However, complementing the current state of knowledge and technology, new additives to cement mortars such as triclosan, hypochlorous acid, silver nanoparticles and graphene oxide have been used.

A literature review [8] has shown that microparticles added to the cement structure fill voids in the porous structure, thereby influencing the increase in strength.

Structural studies showed that samples with hypochlorous acid had the fewest nanopores and the best mechanical properties. Possible effects of hypochlorous acid on concrete can involve reaction with calcium hydroxide (Ca(OH)_2_). Calcium hydroxide, a byproduct of cement hydration, is a relatively weak component of concrete, prone to leaching and chemical degradation. The addition of a small amount of hypochlorous acid may: (i) neutralize a portion of the calcium hydroxide and (ii) produce insoluble compounds (e.g., silicate salts), reducing the porosity of concrete and improving its density and mechanical strength.

As far as pore structure refinement is concerned, hypochlorous acid can lead to the formation of denser calcium silicate hydrates (CSH), which are the primary phase responsible for the mechanical strength of concrete. Reducing porosity enhances its mechanical properties. In terms of activation of pozzolanic additives the acid may facilitate the reaction of pozzolanic materials (e.g., fly ash or silica fume) with calcium hydroxide, resulting in the formation of additional binding products.

The addition of graphene oxide also led to an increase in the strength properties of the cement mortar. Similarly, the largest contact angle was observed for samples with hypochlorous acid and graphene oxide. FTIR and reflectance studies did not show significant differences after adding the additives. This may be due to the small amount of additives that are difficult to detect. However, interestingly, from a bacteriological and fungicidal perspective, the best functional properties were observed in samples with the addition of triclosan and silver nanoparticles.

Triclosan is widely known as an antimicrobial agent with both antibacterial and antifungal activity. Interestingly, it is also used in self-disinfecting paints, which protect against the colonization of microorganisms on wall surfaces [27]. In the presented research, the lowest percentage of viability in S. aureus was observed in sample C + Tri, but for P. aeruginosa, the most limiting factor was C + GO + Ag. These results are not surprising given the greater resistance of Gram-negative bacteria to triclosan, especially since Pseudomonas bacteria are characterized by the presence of multiple mechanisms for the substance’s efflux from cells [28]. Graphene oxide (GO) is a material that is used as a carrier for various substances due to its structure and ease of functionalization [29]. Combining it with nanoparticles, which have antibacterial properties, can result in a synergistic effect, enhancing the effectiveness of the action [30]. Due to the structure of Gram-negative bacteria and their thin cell wall, silver may penetrate the cells more easily and cause internal damage, making Gram-negative bacteria more susceptible to silver nanoparticles [31]. Silver nanoparticles are also effective against Fusarium fungi, including fungal development [32]. This phenomenon explains the lack of fungal growth in our samples doped with silver nanoparticles. This is especially important because Fusarium fungi can occur in public buildings as well as in airconditioned rooms [33,34].

## 5. Conclusions

The conducted studies allowed for the formulation of the following conclusions:

1. The smallest amount of micropores was observed on the surface of the C + HClO and C + HClO + Tr samples, suggesting that these of additives may improve the density of the cement mortar structure.
2. FTIR spectroscopy studies showed that most of the prepared samples (C, C + Ag, C + Tr, C + GO + HClO, C + HClO*, C + GO + Tr, C + GO + Ag, C + HClO, C + HClO + Tr) did not have additional peaks indicating structural changes due to the addition of additives. Similarly, reflectance spectra did not show additional peaks associated with such changes, and the results were similar to the control samples (C, C + GO, C + HClO, C + Tr, C + HClO*, C + GO + HClO, C + Tr + GO, C + GO + Ag, C + HClO + Tr, C + HClO + Ag, C + Tr + Ag, C + GO + HClO + Tr + Ag). This means that the additives did not significantly affect the chemical structure of the material, and changes in its mechanical and biological properties may result from other mechanisms.
3. An increase in strength was observed for the samples doped with hypochlorous acid (C + HClO) and graphene oxide with hypochlorous acid (C + GO + HClO). The flexural strength with a single-point load and compressive strength increased by 18.2–29% compared to the control samples. The increase in strength may be due to the filling of micropores and improvement of the internal structure of the cement mortar thanks to the additives.
4. The greatest inhibition of *Staphylococcus aureus* growth was observed in the C + HClO + Ag sample, while for *Pseudomonas aeruginosa*, the C + HClO and C + HClO + Tr samples exhibited the best results. This suggests that the combination of hypochlorous acid and silver has particularly strong antibacterial activity.
5. The lowest viability of *Staphylococcus aureus* was observed for the C + Tr sample, while for *Pseudomonas aeruginosa*, the C + GO + Ag sample showed the most significant reduction. This result indicates a higher effectiveness of these additives against Gram-negative bacteria, especially due to the synergistic effect of silver and graphene oxide.
6. A complete inhibition of fungal growth was observed in most samples, specifically C + Ag, C + Tr, C + GO + HClO, C + GO + Tr, C + GO + Ag, C + HClO + Ag, C + Tr + Ag, C + GO + HClO + Tr + Ag. This indicates that the addition of silver, triclosan, and graphene oxide is effective in combating fungi, which is important for preventing their growth in damp building conditions.

## 6. Patents

P.447585—The way the grout is made, P.447586—Method of making a hypochlorous acid-based grout.

## Figures and Tables

**Table 1:** Determination of the sample with the composition and concentration of the mixture.

Lp.	Sample Determination	Composition of the Mixture
1	C	Control as cement mortar
2	C + GO	1 kg cement mortar + graphene oxide (concentration 4.5 g/L, 2.75 mL added)
3	C + HClO	1 kg cement mortar + hypochlorous acid (concentration 2000 ppm, 12.8 mL added)
4	C + HClO*	1 kg cement mortar with hypochlorous acid (no water added) (concentration 2000 ppm)
5	C + Tr	1 kg cement mortar + triclosan (concentration 0.0108 g/L, 0.5 mL added)
6	C + Ag	1 kg cement mortar + silver nanoparticles (concentration 100 mg/L, 63 mL added)
7	C + GO + HClO	1 kg cement mortar + graphene oxide (concentration 4.5 g/L, 2.75 mL added) + hypochlorous acid (concentration 2000 ppm, 25.6 mL added)
8	C + GO +Tr	1 kg cement mortar + graphene oxide (concentration 4.5 g/L, 2.75 mL added) + triclosan (concentration 0.0108 g/L, 0.5 mL added)
9	C + GO + Ag	1 kg cement mortar + graphene oxide (concentration 4.5 g/L, 2.75 mL added) + silver nanoparticles (concentration 100 mg/L, 63 mL added)
10	C + HClO + Tr	1 kg cement mortar + hypochlorous acid (concentration 2000 ppm, 12,8 mL added) + triclosan (concentration 0.0108 g/L, 0.5 mL added)
11	C + HClO + Ag	1 kg cement mortar + hypochlorous acid (concentration 2000 ppm, 12.8 mL added) + silver nanoparticles (concentration 100 mg/l.63 mL added)
12	C + Tr + Ag	1 kg cement mortar + triclosan (concentration 0.0108 g/L, 0.5 mL added) + silver nanoparticles (concentration 100 mg/l.63 mL added)
13	C + GO + HClO+ Tr + Ag	1 kg cement mortar + graphene oxide (concentration 4.5 g/l, 2.75 mL added) + hypochlorous acid (concentration 2000 ppm, 12.8 mL added) + triclosan (concentration 0.0108 g/L, 0.5 mL added) + silver nanoparticles (concentration 100 mg/l.63 mL added)

**Table 2:** Contact angle of ultrapure water droplets on the surface of samples C, C + GO, C + HClO, C + HClO*, C + Tr, C + Ag, C + GO + HClO, C + GO +Tr, C + GO + Ag, C + HClO + Tr, C + HClO + Ag, C + Tr + Ag and C + GO + HClO+ Tr + Ag.

Lp.	Determination of the Sample	Contact Angle	Standard Deviation	Comments/Additional Remarks
1	C	54	9	The droplets persisted on the surface and were not absorbed by the grain structure of the centrifugal mortar.
2	C + GO	41	6	
3	C + HClO	55	9	
4	C + HClO*	33	8	After 1 min, all drops were absorbed and the contact angle was 0 deg.
5	C + Tr	50	4	The droplet stayed on the surface and was not absorbed by the grain structure of the centric mortar.
6	C + Ag	56	11	
7	C + GO + HClO	82	20	
8	C + GO + Tr	10	1	The droplet was gradually absorbed after application. After about 15 s, the contact angle was 1–2 deg and the surface was superhydrophilic
9	C + GO + Ag	48	1	The droplet was gradually absorbed after application. After approx. 15–20 s, the contact angle was 0 deg and the surface was superhydrophilic
10	C + HClO + Tr	52	4	The drops persisted on the surface and were not absorbed by the granular structure of the centre mortar.
11	C + HClO + Ag	30	1	The droplet was gradually absorbed after application. After approx. 40 s, the contact angle was 0 deg and the surface was superhydrophilic

**Table 3:** Flexural and compressive strength of cement mortar for samples C, C + GO, C + HClO, C + HClO*, C + Tr, C + Ag, C + GO + HClO, C + GO + Tr, C + GO + Ag, C + HClO + Tr, C + HClO + Ag, C + Tr + Ag, and C + GO + HClO + Tr + Ag.

	Single-Point Bending				Compressive Strength			
Oznaczenie Próbki	Breakload [kN]	Standard Deviation	Strenght [MPa]	Standard Deviation	Breakload [kN]	Standard Deviation	Strenght [MPa]	Standard Deviation
C	0.66	0.02	1.73	0.05	3.70	0.01	2.30	0.01
C + GO	0.68	0.02	1.77	0.06	4.15	0.35	2.60	0.28
C + HClO	0.80	0.01	2.05	0.07	4.65	0.38	2.90	0.21
C + HClO*	0.53	0.03	1.43	0.06	2.93	0.50	1.83	0.31
C + Tr	0.68	0.07	1.75	0.17	3.80	0.17	2.37	0.06
C + Ag	0.63	0.07	1.63	0.21	4.25	0.21	2.70	0.14
C + GO + HClO	0.78	0.01	2.00	0.01	4.73	0.67	2.97	0.38
C + GO +Tr	0.57	0.01	1.45	0.07	4.30	0.14	2.70	0.14
C + GO + Ag	0.50	0.03	1.27	0.06	3.25	0.07	2.05	0.07
C + HClO + Tr	0.71	0.02	1.85	0.07	4.15	0.35	2.60	0.28
C + HClO + Ag	0.56	0.04	1.43	0.12	3.97	0.32	2.50	0.17
C + Tr + Ag	0.42	0.03	1.05	0.07	3.23	0.38	2.03	0.21
C + GO + HClO+ Tr + Ag	0.38	0.03	0.95	0.07	2.65	0.07	1.65	0.07

## Data Availability

The original contributions presented in this study are included in the article. Further inquiries can be directed to the corresponding author.
